# Deregulation of Type I IFN-Dependent Genes Correlates with Increased Susceptibility to Cytomegalovirus Acute Infection of Dicer Mutant Mice

**DOI:** 10.1371/journal.pone.0043744

**Published:** 2012-08-20

**Authors:** Eleonore Ostermann, Lee Tuddenham, Cecile Macquin, Ghada Alsaleh, Julie Schreiber-Becker, Melanie Tanguy, Seiamak Bahram, Sebastien Pfeffer, Philippe Georgel

**Affiliations:** 1 Université de Strasbourg, EA 4438 Laboratoire d'Immunogénétique Moléculaire Humaine, Strasbourg, France; 2 Université de Strasbourg, Architecture et Réactivité de l′ARN, Institut de Biologie Moléculaire et Cellulaire du CNRS, Strasbourg, France; 3 Université de Strasbourg, EA 4438 Laboratoire de Physiopathologie des arthrites, Illkirch, France; Tufts School of Medicine, United States of America

## Abstract

Regulation of gene expression by microRNAs (miRNAs) is now considered as an essential mechanism for cell development and homeostasis. Indeed, numerous studies have reported that modulating their expression, maturation, or activity can affect cell survival, identity or activation. In particular, miRNAs are key players in the tight regulation of signaling cascades, and as such, they appear as perfectly suited immunomodulators. Several immune-related processes, including inflammation, have recently been demonstrated to require specific miRNAs. In addition, the discovery of herpesvirus-encoded miRNAs has reinforced this assumption. To decipher the potential roles of miRNAs in innate antiviral immune response, we developed an *in vivo* model based on the inoculation of mouse cytomegalovirus (MCMV) in mice. Furthermore, we exploited a mouse line carrying a hypomorphic mutation in the *Dicer* gene to visualize the impact of impaired miRNA biogenesis upon the anti-MCMV response. Our data indicate that miRNAs are important actors in mounting an efficient response against herpesviruses. We suggest that a rapid and transient interferon response following viral infection requires miRNA-dependent repressor release. In addition, our *in vivo* efforts identified several miRNA targets, thus providing a conceptual framework for future analyzes on the regulation of specific actors involved in the Type I interferon pathway.

## Introduction

MicroRNAs (miRNAs) are short (22-nt long) non coding RNAs, which are essential regulators of gene expression in multicellular organisms [1]. Many reports have led to the description of a now well-defined pathway whereby genes encoding miRNAs, following RNA polymerase II-mediated transcription, give rise to long primary precursors (pri-miRNAs) that are processed by the nuclear RNase III Drosha. The resulting precursor molecules (pre-miRNAs), which adopt a stem-loop structure, are then exported to the cytoplasm where they are processed by another RNase III (Dicer) to generate double-stranded miRNA intermediates. One strand of this duplex is then incorporated into an Argonaute-containing RNA-induced silencing complex (RISC), resulting in the translational repression and/or degradation of their target mRNAs [2]. Recent data indicate that vertebrates express several hundred miRNAs (741 in *Mus musculus*, 1527 in *Homo sapiens*) [3], each of which can potentially regulate tens to hundreds of target genes. Therefore, the current model stipulates that miRNAs are major modulators of gene expression, and estimates indicate that as many as 60% of all human genes are subject to miRNA-mediated post-transcriptional regulation [4], [5].

It is now widely recognized that cellular miRNAs are implicit in the regulation of many physiological processes, including embryonic development, and the tight homeostasis of immune cells [1]. Furthermore, it is also clear that their misexpression can lead to inappropriate responses to external stimuli such as viruses. Indeed, it was reported in several instances and using different experimental models, that blocking specific miRNAs or decreased miRNA production leads to impaired antiviral defense [6], [7]. Further complexity was unveiled following the discovery of virus-encoded miRNAs in the genomes of large DNA viruses such as herpesviruses [8], [9], [10].

To analyze the contribution of miRNAs to host defense against herpesviruses, we used a model system, namely the experimental infection of mice by murine cytomegalovirus (MCMV), which has previously proved its reliability to study innate antiviral mechanisms such as, for example, Toll-like receptor signaling [11], [12], [13]. We also took advantage of the availability of a mouse strain that carries a hypomorphic mutation in the *Dicer* gene to manipulate miRNA production. This mouse strain enables the analysis of the consequences of reduced Dicer expression in every cell of the animal, as opposed to a targeted deletion in specific tissues using the Flox/Cre system. Using this mutant mouse, it was previously shown that cellular miRNAs play an important role in the defense against vesicular stomatitis virus (VSV) [6]. Here, we further developed this murine model to decipher the complex host-pathogen interaction during acute MCMV infection. We performed both *in vivo* acute infection and *ex vivo* infection of primary macrophages followed by a global analysis of interferon (IFN)-dependent gene expression and quantification of miRNAs involved in inflammation/immune processes. Altogether, our data identified biologically relevant miRNA-targeted IFN-stimulated genes. Our data suggest that repressor release is an important event for the rapid transcriptional induction of MCMV-induced, IFN-mediated genes. Furthermore, our results point toward a dominant role of cellular miRNAs as protective factors compared to viral miRNAs, which are usually predicted to carry pathogenic functions.

## Materials and Methods

### Mice

Dicer1-deficient mice (*Dicer^d/d^*) were generated by Jiahuai Han's laboratory and described in [6]. The *Dicer^d/d^* line was backcrossed more than 10 times against C57BL/6 in our laboratory; littermate controls are indicated by +/+ in all figures. Mice used in all experiments were age- and sex-matched. Animals were maintained under pathogen-free conditions in the animal care facility of the Institut d'Immunologie et d'Hématologie.

### Ethic statement

Handling of mice and experimental procedures were conducted in accordance with the French Law for the Protection of Laboratory Animals. The procedures were approved by the service véterinaire de la Préfecture du Bas-Rhin (France) under the authorization number A-67-345 and reviewed by the Regional Ethical Committee for Animal Experimentation (CREMEAS) of the Strasbourg University.

### Cell culture

To harvest peritoneal macrophages, mice were injected intraperitoneally (i.p.) with 3% thioglycolate (TG). After 3 days, peritoneal exudates were harvested in 5 ml of PBS. After centrifugation, cells were suspended in DMEM (Gibco) supplemented with 5% FCS and 2% penicillin-streptomycin. Cells were plated at 2×10^6^ cells per well (6 well plates) and incubated at 37°C in a 5% CO2 atmosphere. After removal of non-adherent cells, adherent cells were washed with PBS and infected at the indicated multiplicity of infection (MOI).

### Viruses

The Perth strain of MCMV was a salivary gland-passaged virus stock prepared in BALB/c mice. MCMV was titrated by plaque assay on M2-10B4 cells and was injected intra peritoneally (i.p.) to mice at doses of 5×10^5^ p.f.u/mouse.

The Smith strains of MCMV expressing Luciferase (MCMV-Luc) or Cre (MCMV-Cre), a kind gift of Ulrich Koszninowski, were obtained by BAC mutagenesis and amplified in M2-10B4 cells [14]. MCMV-Luc was injected i.p. at 5×10^4^ p.f.u/mouse.

### IFN PCR array

Total RNA was extracted from approximately 4×10^6^ peritoneal macrophages or spleens with RNeasy mini kits (Qiagen) with inclusion of a DNase I treatment step. RNA quality was verified by Agilent 2100 Bioanalyzer profiles.

cDNA was prepared from 1 µg (macrophages) or 2 µg (spleen) of total RNA by using a RT^2^ PCR array first strand kit. Quantitative real time PCR was performed with an ABI PRISM 7000 using Mouse Interferon α, β Response RT^2^ Profiler PCR array (Qiagen, SA biosciences) according to the manufacturer's instructions. Complete datasets are available upon request.

### miRNA array

Total RNA was extracted using miRNeasy mini kits (Qiagen, Courtaboeuf, France). cDNA was prepared from 1 µg (macrophages) or 4 µg (spleen) of total RNA using a RT^2^ miRNA First Strand Kit. Quantitative real time PCR was performed with an ABI PRISM 7000 using Mouse Immunopathology RT^2^ miRNA PCR array (Qiagen, SA biosciences) according to the manufacturer's instructions. Datasets are available upon request.

### Quantitative RT-PCR

Total RNA was isolated from different tissues using either TRIZOL (Invitrogen), or RNeasy Mini Kit with inclusion of a DNase I treatment step (Qiagen). After reverse transcription of 1 μg of total RNA by ImProm-II™ Reverse Transcriptase (Promega), quantification was performed with an ABI PRISM 7000 (Applied Biosystems) using FastStart Universal SYBR Green Master (ROX) (Roche Molecular Diagnostics, Meylan, France). The PCR parameters were as follow: 2 min at 50°C, 10 min at 95°C followed by 40 cycles of denaturation at 95°C for 15 s, and annealing and extension at 60°C for 1 min. Amplification was performed in a final volume of 20 μl, and each sample contained 2 μl cDNA (equivalent to 10ng of RNA), 500 nM of forward and reverse primers and 10 μl of FastStart Universal SYBR Green Master (ROX) (Roche Molecular Diagnostics). Each quantitative PCR reaction was performed in triplicate, and the mean Ct value for each sample was used for data analysis. Samples were normalized to β-actin, results are expressed as fold changes of Ct values relative to controls by using the 2-ΔΔCt formula. Primers used were the following:

dicer 24 F: 5′ AGAACGAAATGCAAGGAATGGA

dicer 25 R: 5′ CTTCTTTCTCCTCATCCTCCTCG

Amplicon size: 65 bp

irf7 F: 5′ CCCATCTTCGACTTCAGCAC

irf7 R: 5′ TGTAGTGTGGTGACCCTTGC

Amplicon size: 79 bp

actin F: 5′ AGAGGGAAATCGTGCGTGAC

actin R: 5′ CAATAGTGATGACCTGGCCGT

Amplicon size: 139 bp

cxcl10 F: 5′ CTGCTGGGTCTGAGTGGGACT

cxcl10 R: 5′ CCTATGGCCCTCATTCTCACTG

Amplicon size: 101 bp

isg20 F: 5′ GAACATCCAGAACAACTGGCG

isg20 R: 5′ GTAGAGCTCCATTGTGGCCCT

Amplicon size: 67 bp

ifit1 F: 5′ CAACTGAGGACATCCCGAAACA

iIfit1 R: 5′ ATGTGGGCCTCAGTTTCAAAGT

Amplicon size: 109 bp

oas1a F: 5′ CCAAGGTGGTGAAGGGTGG

oas1ar: 5′ ACCACCAGGTCAGCGTCTGA

Amplicon size: 73 bp

PRIMER EXPRESS software Applied Biosystems) was used to design the primers.

### Absolute Quantitative PCR of MCMV genome

DNA was extracted from different tissues using the phenol-chloroform method. Primers cmv IE1 F (5′-ACTAGATGAGCGTGCCGCAT) and mcmv IE1 R (5′-TCCCCAGGCAATGAACAATC) were chosen to amplify a segment of exon 4 of the IE1 gene of MCMV, and a cellular gene (encoding Zinc-α1 glycoprotein, zag) was detected with primers zag Forw. (5’TTCAGGGAATGTTTGGTTGCG) and zag Rev.(5′TGAAATCCTCTCCGTCGTAGGC). Serial dilutions of pGEM-Teasy (Promega) expressing MCMV ie3/4, and of pCR2 expressing zag [15] were used as standards to determine the MCMV genome copy number, and the number of cells respectively. No-template controls, and DNA from uninfected mice served as negative controls. The PCR parameters were as above except that 100 ng of DNA was used as template. Mean Ct values were used for data analysis; n = 3.

### Northern blot analysis of miRNAs

Northern blotting of miRNAs was performed as described previously [16] using 2 µg of total RNA and radiolabeled antisense oligodesoxyribonucleotides complementary to miR-16, miR-M23-2 or part of U6 snRNA as probes. Northern blots were exposed overnight to phosphorimager plates (Fuji) and scanned using a FLA-5000 series phosphorimager (Fuji).

### Cell lysis and immunoblotting procedures

Tissues were homogenized (Omni Tissue Master) and proteins were isolated in lysis buffer (150 mM NaCl, 50 mM Tris–HCl, pH 8, 0.5 mM EDTA, 1% NP-40, 10% glycerol) supplemented with complete protease inhibitor (Roche Applied Science) and sodium orthovanadate (1 µM) (Sigma). Lysates were incubated on ice for 20 min followed by centrifugation at 4°C for 15 min at 13,000 rpm; supernatants were kept at −20°C for further analysis. Total protein concentration was determined using the Bradford Assay (Bio-Rad). Samples (40 μg) were mixed with Laemmli buffer, boiled for 5 min, and then separated on a 10% polyacrylamide gel (Bio-Rad) followed by semi-dry blotting onto Hybond-C Extra membranes (Amersham Pharmacia Biotech). Proteins were revealed with anti-GAPDH (Millipore), anti-IRF7, anti-Stat1α p91 (Santa-Cruz Biotechnology) using an HRP-goat anti-mouse or HRP-goat anti-rabbit IgG (H + L) (Bio-Rad) as secondary antibodies. Blots were developed with the ECL system (Amersham Pharmacia Biotech).

### Quantification of cytokines

IFN-β or CXCL10 in serum and cell supernatants were measured with Mouse Interferon Beta ELISA Kit (PBL InterferonSource) and Mouse CXCL10 ELISA kit (R&D Systems) following the manufacturer's instructions.

### Flow cytometry

Spleen cell suspensions were incubated 2 h with brefeldin A (10 µg/ml) before staining with FITC-conjugated anti-NKp46 (R&D Systems). For intracellular staining, cells were fixed and incubated with PE-conjugated anti-IFN-γ (BD Pharmingen). Flow cytometry was performed by using a FACSCalibur flow cytometer (BD Biosciences) and analyzed with Cellquest Software.

### Luciferase reporter constructs

To generate luciferase-based reporter plasmids, psiCHECK-2 (Promega) was modified by inserting the Gateway cassette C.1 (Invitrogen) at the 3′ end of the firefly luciferase gene (f-luc) into the Xba *I* site of psiCHECK-2. The 3′ untranslated region (UTR) of Cxcl10 was amplified from NIH-3T3 genomic DNA. After PCR addition of attB1 and 2 sequences, the resulting products were cloned into pDONR/Zeo and then recombined in the modified psiCHECK-2 vector using Gateway technology (Invitrogen). The following primer sequences were used (sense and antisense primers are indicated in this respective order; anchor sequences used for the nested PCR are underlined):

Cxcl10: 5′ AAAAAGCAGGCT TCCGCTCAATACAGTTTCCTC

and 5′ AGAAAGCTGGGT CTACCCACCCCAACTTCTTG

attB1/2: 5′ ACAAGTTTGTACAAAAAAGCAGGCT

and 5′ ACCACTTTGTACAAGAAAGCTGGGT.

Mutagenesis of the predicted miR-21 binding site was performed as described previously [17] using mutagenesis primers as follows (the mutated nucleotides are underlined):

Sense: 5′ TCCTAGCTCTGTACTGTATCGTATGTGGAGGTGCGACGC 3′

Antisense: 5′ GCGTCGCACCTCCACATACGATACAGTACAGAGCTAGGA 3′.

### Transfections and luciferase assay

HeLa cells (2.5×10^4^) were seeded in 48-well plates in antibiotic-free medium with tiny LNA inhibitors (1 µM) against miR-21, or to that of a negative control miRNA (cel-miR-67). Transfections were performed following 24 h of treatment using 0.5 µl Lipofectamine 2000 (Invitrogen), and 50 ng dual luciferase reporter plasmids. At 48 h post transfection, cells were washed with PBS and lyzed in 65 µl of Passive Lysis Buffer (Promega). Firefly and Renilla luciferase activities were measured from 10 µl of each lysate using the dual luciferase reporter Assay System (Promega) and a luminometer (Glomax, Promega).

### *In vivo* luminescence assay

Eight hours after intraperitoneal MCMV-Luc infection, animals were anesthetized (10 mg/ml ketamine and 2 mg/ml xylazine), hairs were removed by chemical depilation, and mice received a first i.p. injection of Luciferine (150 mg/kg body weight). Luminescence was quantified using the In Vivo Imaging System (IVIS) from Xenogen/Caliper. Afterwards, similar Luciferin injections and light emission recording were performed at Days 1, 2, 3 and 4. Luminescence was expressed as a percentage to that quantified 8 hours post-infection.

### Statistical analysis

Student's t (two-tailed unpaired) test was used to compare two independent groups using GraphPad software. A probability (p) value of < 0.05 was considered to be significant. *p < 0.05; **p < 0.01; ***p < 0.001.

LogRank test was used for statistical analysis of survival experiments.

## Results

### 1. A subset of Type I IFN-regulated genes is overexpressed in Dicer-deficient splenocytes and macrophages

**Figure 1:**
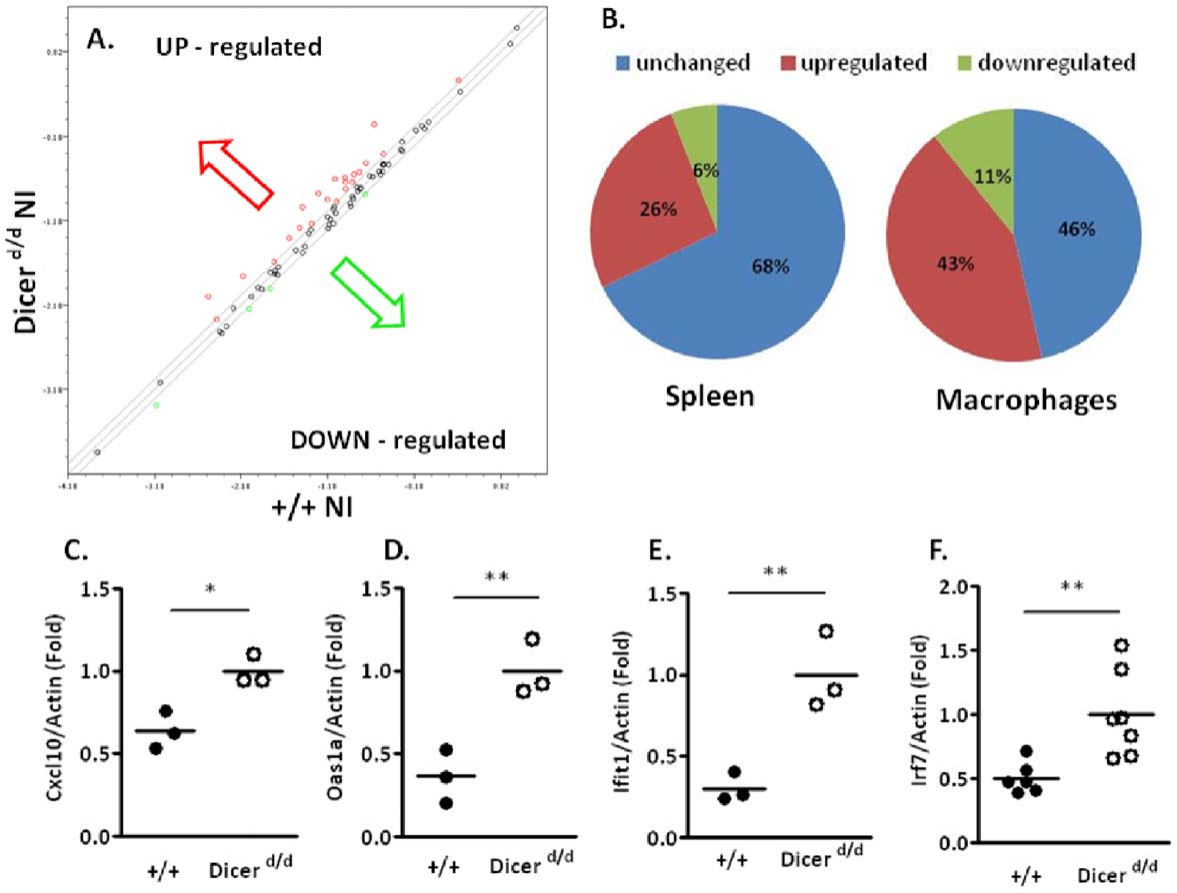
Dicer-deficient mice exhibit constitutive up-regulation of IFN-stimulated genes in splenocytes. A. Scatterplot illustrating macroarray data performed on mRNA from non-infected (NI) spleens harvested from control mice (+/+) or *Dicer^d/d^* mutants. Red dots indicate up-regulated genes in mutants, whereas green dots represent down-modulated genes. B. Repartition of up-regulated genes (red), down-regulated (green), or whose expression remains similar in *Dicer^d/d^* splenocytes and macrophages compared to controls (blue). C–F. qRT-PCR experiments on selected IFN-dependant genes performed on mRNA extracted from control (+/+) or mutant (*Dicer^d/d^*) spleens.

Because a well-coordinated Type I IFN response is mandatory for mounting an efficient antiviral defense, we first undertook a thorough characterization of this pathway in control and *Dicer^d/d^* animals under steady-state (non-infected) conditions. During its initial characterization, the *Dicer^d/d^* mutant was shown to be hypersusceptible to vesicular stomatitis virus (VSV) infection, independently from Type I IFN perturbation [6]. However, only a few elements (i.e. IFN-β production) of this response were monitored, and since then, miRNA-dependent expression of several genes involved in the IFN pathway has been reported. Therefore, a comprehensive analysis of this signaling cascade appeared necessary. To this end, we used a commercial PCR macroarray to follow the expression pattern of a panel of 84 genes involved in the IFN pathway at several levels: signaling molecules, receptors, signal transduction and target genes. Interestingly, we observed augmented expression of many (22/84; 26%) genes in *Dicer^d/d^* splenocytes compared to control cells (Fig. 1A). In contrast, only 6 genes were downregulated in mutant cells. We replicated this analysis in peritoneal macrophages (a more homogenous cell population than splenocytes), and observed that a larger (43%) proportion of IFN-related genes were upregulated (Fig. 1B).

Looking more carefully at the complete gene list in both systems, we noticed that the transcription of only 8 genes (Table 1) was significantly upregulated (p<0.05) in mutant splenocytes. Among these, *Irf7, Cxcl10, Oas1a* and *Oas2* were also upregulated in macrophages. To confirm the upregulation of these candidate genes, we performed an independent qRT-PCR analysis on mRNA extracted from spleens and macrophages from additional wild-type and *Dicer^d/d^* mice. As shown in Figure 1C–F, statistically significant increases in expression were observed in every case, validating our macroarray data. We also analyzed the expression of Dicer in these samples to verify that the previously described mutation actually lowers its accumulation. We found that Dicer mRNA was strongly downregulated (up to 80%) in both *Dicer^d/d^* spleen cells and macrophages. Low *Dicer* expression in mutants compared to wild-type was observed in every organ tested (lungs, brain, salivary glands; data not shown). These observations were confirmed at the protein level by western blot from whole spleen isolates (not shown).

**Table 1:** Significantly induced genes in *Dicer^d/d^* splenocytes.

*Genes*	Fold	p value
*Cxcl10*	1.4832	0.042309
*Gbp1*	1.387	0.000552
*Ifit1*	2.7934	0.00814
*Ifitm1*	1.7435	0.024338
*Irf7*	2.5823	0.016564
*Irf9*	1.3806	0.039564
*Oas1a*	2.6982	0.004392
*Oas2*	2.9869	0.023864

### 2. MicroRNA profiling in splenocytes from naïve *Dicer^d/d^* mice shows specific miRNA alteration

**Figure 2:**
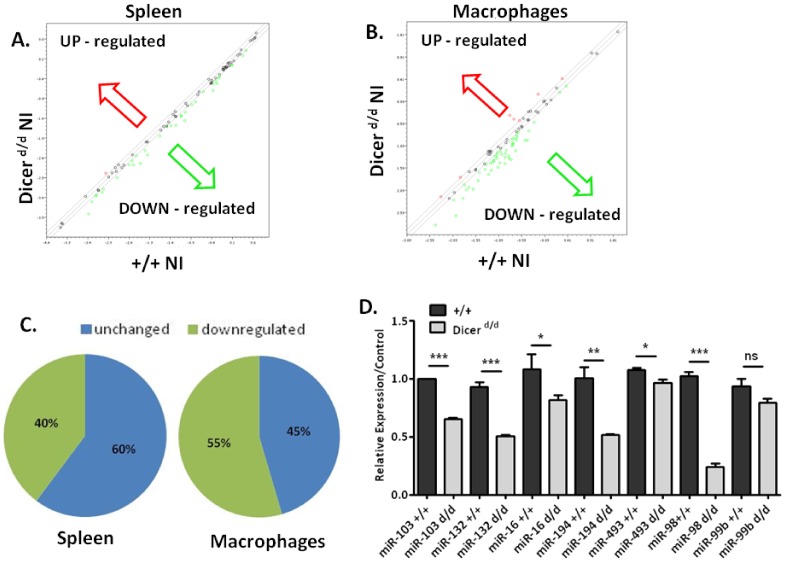
Impaired maturation of a subset of miRNAs in *Dicer^d/d^* mutant splenocytes and macrophages. A. Scatterplot showing relative expression of miRNAs by macroarray. In red, are shown miRNAs up-regulated in naïve (NI) splenocytes from *Dicer^d/d^* mutants and green depicts down-regulated miRNAs. B. Scatterplot obtained upon macroarray analysis of mRNA extracted from control (+/+) and *Dicer^d/d^* non-infected macrophages. C. Repartition of miRNAs down-regulated (green) or with similar expression (blue) in mutant splenocytes and macrophages. D. qRT-PCR experiments on selected miRNAs were performed on miRNA isolated from control (+/+, dark bars) and *Dicer^d/d^* mutant (white bars) spleens.

Having identified several Dicer-dependent candidate genes in the IFN pathway, we next monitored the expression profile of miRNAs known to be associated with inflammatory/immune processes from the literature. We monitored 88 such miRNAs spotted on a commercial PCR macroarray. As can be seen in Fig. 2A, we observed a reduction in miRNA expression in splenocytes of *Dicer^d/d^* mutants. Interestingly however, only 40% of the tested miRNAs were affected by the mutation (downregulated by at least 30%). Despite a very significant alteration of *Dicer* expression, the amount of most (60%) miRNAs remained similar (± 1.3 fold) in Dicer mutant splenocytes (Fig. 2C). The proportion of miRNAs presenting a significantly decreased expression is slightly more important (55%) in macrophages (Fig. 2B–C).

To validate our macroarray data, we performed qRT-PCR on selected miRNAs (Fig. 2D). These experiments confirmed that the expression of highly affected miRNAs (such as miR-98) is indeed significantly reduced (80%) in *Dicer^d/d^* spleen cells. From the PCR array data, we extracted a list of miRNAs whose expression was repressed >1.5– fold in mutant spleen cells (Table 2). Remarkably, we observed that many miRNAs within that list, were also predicted by the miRanda program [18] to target the mRNAs showing significantly increased expression (listed in Table 1). Thus, Cxcl10 and miR-210, miR135a/b, or Oas2 and miR-7d, miR-140, miR-98, let-7f, displayed opposite regulation in Dicer mutant cells, and may be involved in the same networks *in vivo*. In addition to *Cxcl10* and *Oas2*, *Ifit1* and *Gbp1* could also be direct targets of miRNAs that are listed in Table 2. Other upregulated genes such as *Irf7* possess a very short 3’UTR, which is incompatible with miRNA targeting, unless they contained binding sites within their coding sequences, which we have not explored. In addition, none of the miRNAs predicted to regulate Irf9, Oas1a or IfitM1 mRNAs (i.e. miR-20, −23, −106a) exhibited noticeable downregulation in *Dicer^d/d^* splenocytes. Therefore, we hypothesized that a miRNA-dependent transcriptional regulator might represent a common signature to these genes that would explain their coordinated overexpression. In order to identify such a factor, we used the SABiosciences' proprietary database (DECODE, DECipherment Of DNA Elements) to search for binding sites for the most relevant transcription factors in the promoters of the corresponding human genes (for which data on transcriptional regulation are more abundant and curated compared to mouse genes). We found that the four above-mentioned genes contain binding motifs for STAT1 in their promoters. To get an insight into the validity of this prediction, we compared the expression pattern of all genes spotted on the macroarray according to their potential STAT1-dependence. A highly significant difference (p < 0.0006) was observed when we plotted and compared the fold regulation of STAT1-dependent genes *vs*. STAT1-independent genes (Fig. S1). In parallel, we compared fold induction of genes containing, or lacking, binding sites for an unrelated transcription factor, AP1, in their promoters; the 2 groups appeared statistically undistinguishable (data not shown). This indicates that Stat1 mRNA could be controlled in a miRNA-dependent manner, and indeed, the miRanda algorithm predicts potential binding sites in its 3’ UTR for numerous miRNAs including miR-214, miR-194 and miR-140, which are listed in Table 2. An alternative hypothesis for differential gene expression in Dicer^d/d^ splenocytes compared to wild-type could reside in a modification in the proportion of the cellular content if this organ. However, we do not favor this option because our quantification by flow cytometry of different splenic cell populations (NK, Dendritic cells, CD8+ and CD4+ T cells) did not reveal marked changes between controls and mutants (not shown).

**Table 2:** List of miRNAs strongly affected by the mutation in *Dicer^d/d^* splenocytes.

Mature ID	Fold Regulation
miR-135b	−2.6965
miR-363	−2.5995
miR-98	−2.543
miR-132	−2.355
miR-103	−2.1776
miR-99b	−2.044
miR-135a	−1.8734
let-7d	−1.7861
miR-130a	−1.6538
miR-152	−1.6246
miR-129-5p	−1.6232
miR-298	−1.6169
miR-185	−1.6035
miR-214	−1.5746
miR-140	−1.5688
miR-134	−1.5667
miR-18b	−1.5607
miR-194	−1.5509
let-7f	−1.5107
miR-149	−1.51

### 3. Potential target validation at the protein level

**Figure 3:**
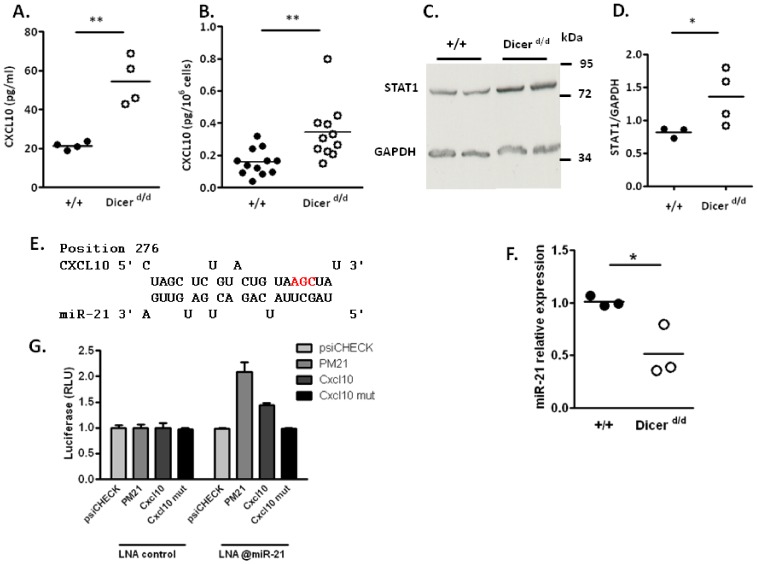
Increased protein expression of putative miRNA-targeted genes in *Dicer^d/d^* mutants. A. CXCL10 was quantified in the serum of naïve controls (black dots) and Dicer mutant mice (white dots). B. CXCL10 (expressed in picograms for 1×10^6^ cells) quantification in the supernatant of macrophages harvested from wild-type (+/+) and *Dicer^d/d^* mutants. C. STAT1 expression visualized by Western blot performed on spleen extracts from two control animals and two *Dicer^d/d^* mutant mice. GAPDH serves as loading control. D. Quantification of STAT1 signals in spleen protein extracts normalized to GADPH was done for 3 controls and 4 mutants. E. Sequence alignment of murine miR-21 and its target sequence in the Cxcl10 3’UTR. The mutated nucleotides disrupting miR-21 binding in the mutated Cxcl10 3’UTR are shown in red. F. miR-21 expression is reduced in Dicer d/d splenocytes compared to wild-types. qRT-PCR was used to quantify miR-21 expression in wild-type and *Dicer^d/d^* splenocytes. G. Normalized Luciferase expression (RLU) in HeLa cells. Control or anti-miR-21 (@miR-21) tiny LNAs were incubated with HeLa cells for 24 h. Afterward, cells were transfected with empty vector (psiCHECK-2), or vectors containing a perfect match for miR-21 (PM21), the wild-type Cxcl10 3’UTR, or a mutated Cxcl10 3’UTR downstream of the f-luc gene.

Because miRNAs typically regulate translation in animal cells, we compared CXCL10 and STAT1 protein levels in both control and *Dicer^d/d^* animals and cells. We first quantified the expression of circulating CXCL10 in the blood of naïve mice by ELISA, and observed a significant (p<0.01) increase in CXCL10 expression (2-fold) in serum harvested from *Dicer^d/d^* mice (Fig 3A). We also monitored CXCL10 secretion in supernatants from primary splenocytes, and observed increased chemokine production by mutant cells (Fig. 3B). To evaluate STAT1 expression levels, we performed western blots on total protein extracted from control and *Dicer^d/d^* spleens. As shown in Fig 3C, STAT1 protein is overexpressed in mutant spleens compared to wild-type controls, an observation confirmed in several animals (Fig. 3D). Altogether, these data, in conjunction with mRNA and miRNA macroarray profiling suggests that both Cxcl10 and Stat1 mRNAs are subjected to miRNA-dependent regulation *in vivo*. Quantification of Stat1 (one of the 84 genes tested on the array) mRNA was performed in the course of our macroarray experiments. Interestingly, no significant difference was noticed when we compared wild-type and mutant cells (splenocytes or macrophages), indicating that miRNA-dependent STAT1 expression is mostly controlled at the translational level.

### 4. Cxcl10 is a direct miRNA target

To demonstrate direct involvement of miRNAs in the control of Cxcl10 expression, we turned to a luciferase reporter assay. As stated above, the miRanda algorithm predicted binding sites for numerous miRNAs, including miR-210 and miR-135a/b. However, the TargetScan algorithm [19] only predicted binding sites for members of the miR-16 and miR-21 families. In order to confirm that miRNAs are indeed regulating Cxcl10 expression, we decided to focus on miR-21, which is abundantly expressed in the cells (HeLa) we used for luciferase reporter assay. In addition, the putative binding site of miR-21 is more extensive than the miR-16 one as assessed using RNAhybrid [20] (Fig. 3E). Finally, we observed a 2-fold reduction of miR-21 expression in *Dicer^d/d^* mutant compared to wild-type splenocytes (Fig. 3F). We thus constructed a luciferase sensor for Cxcl10 by inserting its full length 3′UTR downstream of the firefly luciferase coding sequence within the psiCHECK-2 plasmid. As a positive control, we also constructed a reporter containing a perfectly matched miR-21 binding site downstream of f-luc. We co-transfected these constructs, or a plasmid containing no insert, in HeLa cells together with tiny LNA oligonucleotides [21] designed to base-pair with the seed of miR-21 or to that of a control miRNA (cel-miR-67). We found that the perfect match sensor, and the Cxcl10 sensor were up-regulated two and 1.5-fold respectively in the presence of the tiny LNA directed against miR-21 (Fig. 3G). Insertion of a three-nucleotide bulge within the predicted miR-21 seed-match in the Cxcl10 sensor abolished this effect.

These data suggest that the Cxcl10 mRNA can be directly targeted for post-transcriptional regulation by miR-21. It remains of course possible that other miRNAs, such as miR-210, miR-135a/b or miR-16, could also be involved in the regulation of this chemokine.

### 5. *Dicer^d/d^* mice exhibit hypersusceptibility to acute MCMV infection

**Figure 4:**
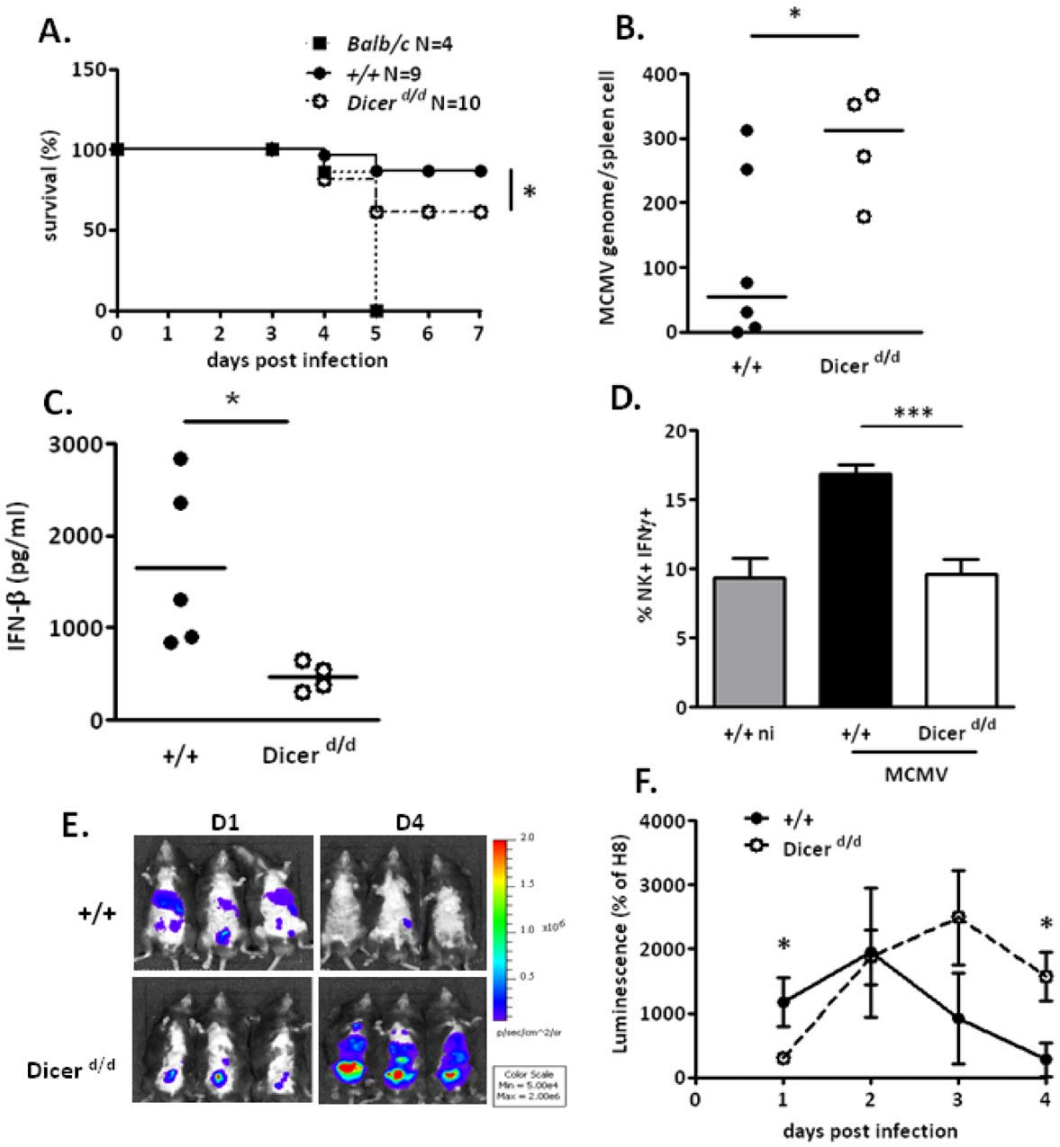
*Dicer^d/d^* mice are hypersusceptible to acute MCMV infection. A. Survival curve of control mice (+/+, black spots), *Dicer^d/d^* (white spots) and BALB/c animals (black squares) upon acute MCMV infection (5×10^5^ pfu/mouse). B. Absolute quantification of the MCMV genome by qPCR in infected wild-type mice (+/+) and *Dicer^d/d^* mutants. C. IFN-β quantification by ELISA in the serum of controls (+/+) and mutants 36 hours post-infection (h.p.i). D. Quantification of activated NK cells (NKp46+/IFN-γ+) in the spleen of non-infected (NI) and infected controls (+/+) and Dicer d/d mice 72 h.p.i. 6 animals/group were used. E. Snapshot images showing MCMV-derived luminescence 1 (D1) and 4 days (D4) upon MCMV-Luc infection of controls (+/+) and *Dicer^d/d^* mice. F. Time-course analysis of the luminescence quantification in MCMV-Luc infected controls (n = 6 +/+; black spots) and mutants (n = 5; dotted line and white spots).

To evaluate the impact of reduced *Dicer* expression on immune responses directed against herpesviruses, we next performed acute MCMV infection of control (^+/+^) and *Dicer^d/d^* mutant mice by injecting intraperitonally 5×10^5^ p.f.u of virus, and monitoring the survival rate of the animals. We initially expected that the constitutively moderate increase in Type I IFN signaling of the Dicer mutant would improve antiviral defense. We were therefore surprised to observe a higher mortality of *Dicer^d/d^* mice upon MCMV challenge (Fig. 4A). The difference in survival rate between *Dicer^d/d^* and control mice was statistically significant (p = 0.0429), but the phenotype clearly diverges from that which is observed in BALB/c mice, which as opposed to C57BL/6 mice, are highly susceptible to MCMV infection due to a mutation in the NK cell receptor ly49H [22], [23]. We therefore suspected a subtly deregulated antiviral response in the mutant, rather than a lack of virus detection. Using absolute viral DNA quantification, we noted increased MCMV replication in *Dicer^d/d^* splenocytes (Fig 4B). To gain more insight into the potential defective response of *Dicer^d/d^* mice to MCMV infection, we characterized and compared their anti-MCMV response to that of control animals. First, we quantified IFN-β in the serum of MCMV-challenged controls and *Dicer^d/d^* mice. As illustrated in Fig. 4C, at 36 hours post-infection (hpi), IFN-β secretion was significantly diminished in the blood of mutant mice. We next monitored NK cell response, the integrity of which is mandatory for effective viral clearance. In line with the lower IFN-β production, we observed less (IFN-γ +) NK cells in the spleens of infected *Dicer^d/d^* mice than in wt mice at 3 days post infection (dpi) (Fig. 4D), reflecting a slightly impaired NK cell response.

In a parallel experiment, we performed intraperitoneal injections of *Dicer^d/d^* and *Dicer^+/+^* mice with a recombinant luciferase-expressing MCMV. Using appropriate imaging equipment to visualize light emission, we were able to follow viral replication *in vivo*. We observed that, one day after viral inoculation, *Dicer^d/d^* mutants seemed to control the infection more effectively than controls (Fig. 4E). This is likely due to the moderate, but constitutive overexpression of IFN-responsive genes in these animals. However at day 4, mutants exhibited a striking hypersusceptible phenotype characterized by higher viral load compared to wild-type animals. We also performed a time-course analysis of the luminescence produced by the virus in the infected mice to further quantify these observations (Fig. 4F).

These data most probably reflect a complex situation in which the maturation of both cellular and viral miRNAs is affected by the mutation. Furthermore, the development of an effective antiviral response requires the construction of a network of immune cells such as macrophages, dendritic cells, and NK cells interconnected by a large panel of cytokines and chemokines (i.e, IL-12, IL-15, Type I IFN, etc), which makes it difficult to tackle the precise origin of this increased viral susceptibility.

### 6. *Ex vivo* infections confirm that cell-autonomous miRNA defect increases susceptibility to MCMV

**Figure 5:**
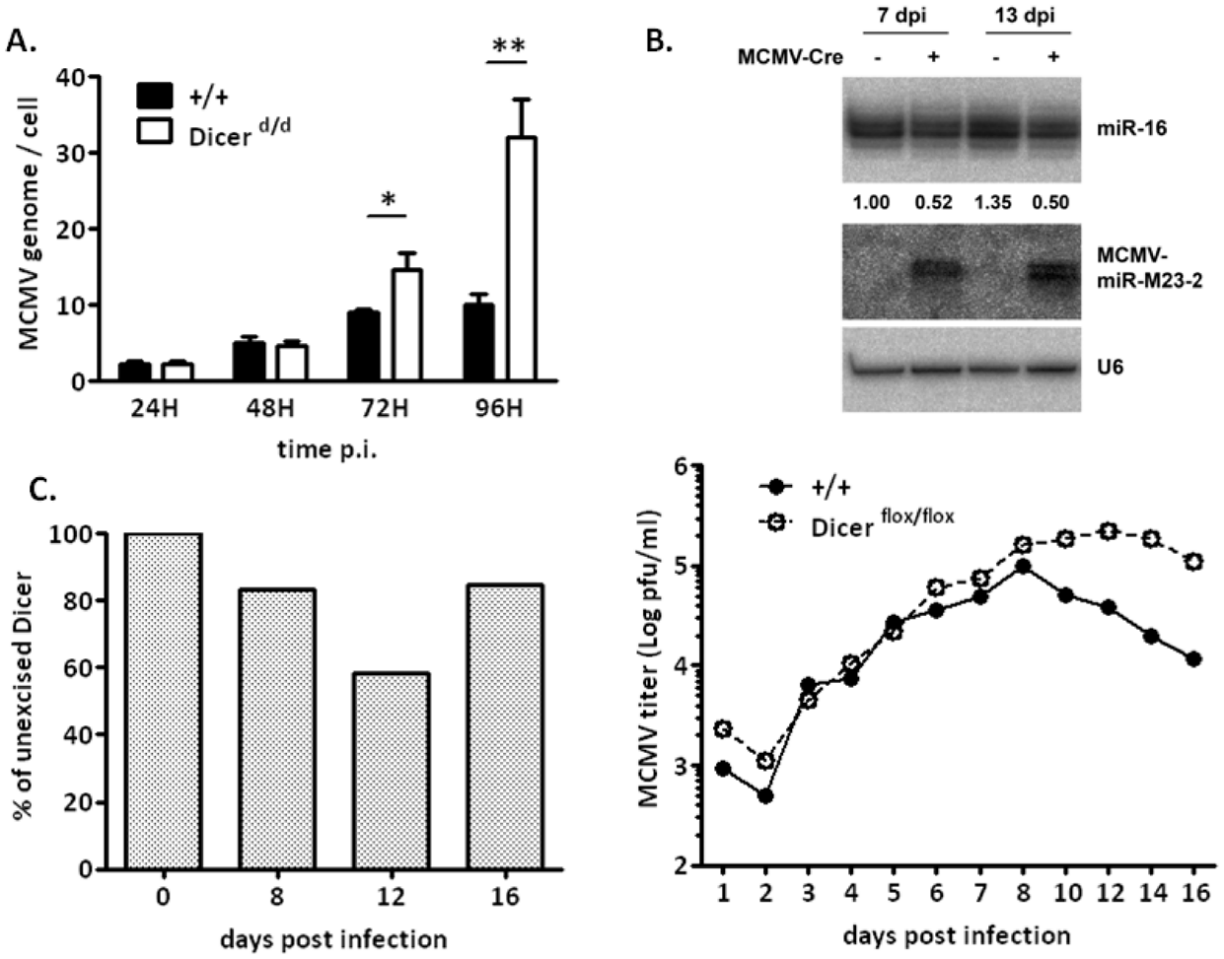
*Ex vivo* infected macrophages harvested from Dicer-deficient mice exhibit antiviral defects. A. Absolute quantification by qPCR of the MCMV genome in control (+/+, black bars) macrophages and cells isolated from *Dicer^d/d^* mice (white bars) at different time points post infection (p.i). B. Northern blot experiments showing, miR-16 and MCMV miR-M23-2 expression in peritoneal macrophages isolated from *Dicer^flox/flox^* mice 7 days and 13 days upon MCMV-Cre infection. Numbers below miR-16 panel indicate relative expression. Controls (Ctrl) are mock-infected macrophages. U6 expression serves as loading control. C. Quantification of Dicer excision efficacy upon MCMV-Cre infection of Dicer-floxed macrophages. Intensity of bands corresponding to excised and non-excised alleles was measured from agarose gels. Ratio is plotted for days 8, 12 and 16. D. Increased viral replication in Dicer-floxed macrophages upon MCMV-Cre infection. MCMV titer was measured by plaque assay in the supernatant of MCMV-Cre infected macrophages harvested from controls (+/+; dark spots) and *Dicer^flox/flox^* mice (white spots).

To decipher in more detail the molecular defects leading to increased MCMV susceptibility of Dicer-deficient animals, we turned to thioglycolate-elicited peritoneal macrophages, which represent a convenient source of semi-purified MCMV-permissive cells. As seen in Fig. 5A, peritoneal macrophages isolated from *Dicer^d/d^* mice cannot efficiently control viral replication, as the amount of MCMV genomes/cell is notably increased in these cells. To confirm these data, we turned to a model of conditional ablation of Dicer in macrophages. For this, peritoneal cells isolated from transgenic mice (in which the *Dicer* gene is surrounded by Lox-P sites, [24]) were infected with a CRE-recombinase-expressing recombinant MCMV [14]. As seen in Fig. 5B, seven and thirteen days post infection with the recombinant virus, cellular miR-16 expression was diminished (1.9-fold at day 7 and 2.6-fold at day 13) reflecting the expected defect in miRNA maturation. Concomitant increase of the precursor form (pre-miR-16) was also observed (data not shown). Interestingly, viral miR-23-2 production can still proceed, which indicates that the remaining DICER is sufficient to process newly expressed miRNAs. To quantify the efficacy of *Dicer* ablation using this system, we used a PCR-based assay, which permits the simultaneous detection of the excised and non-excised alleles. Fluorescence quantification of the bands visualized on the gel was plotted in Fig. 5C. It appears that under such conditions of infection (multiplicity of infection (MOI)  = 1), recombination at the *Dicer* locus is limited (40% efficiency), and reaches its maximum efficacy between day 12 and 16. Improved efficiency was obtained upon infection at an MOI of 10 (data not shown). Nevertheless, under these conditions of partial Dicer mRNA reduction, we also found that MCMV replication is uncontrolled, as viral particle production, quantified by plaque assay, is increased in Dicer-deficient compared to wt cells (5- to 10-fold at 12 and 16 dpi) (Fig. 5D). Altogether, these data indicate that the reduction of Dicer expression in isolated cells affects MCMV replication. Such a cell-autonomous phenotype demonstrates that the increased viral susceptibility observed *in vivo* is not only the result of an impaired immune response, but that other essential mechanisms such as DNA replication control and the production of antiviral proteins are also likely to play a key role.

### 7. Macroarray analysis of IFN-regulated genes in MCMV-infected controls and Dicer-deficient cells suggests the importance of repressor release

**Figure 6:**
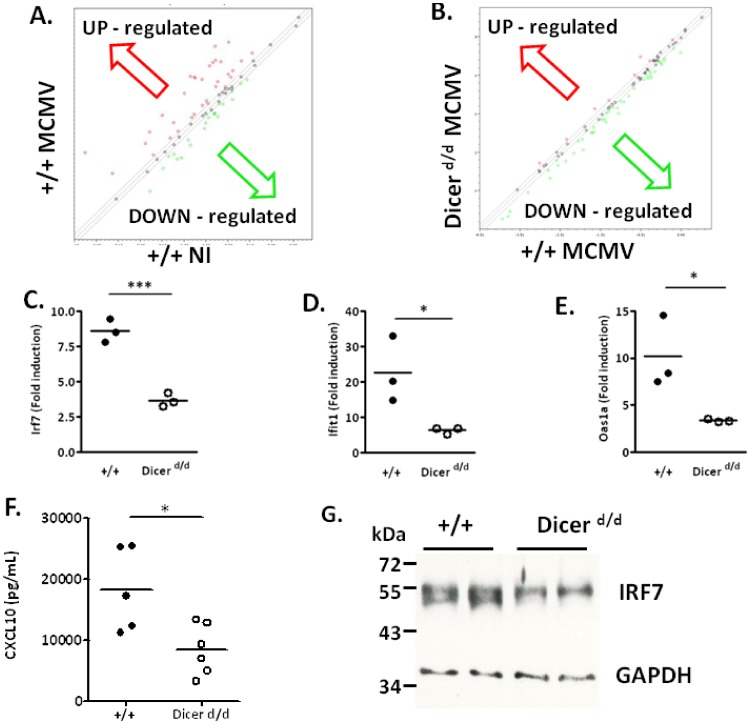
IFN-stimulated genes induction is impaired in MCMV-infected *Dicer^d/d^* mice. A. Scatterplot shows multiple IFN-dependent genes up-regulation (red dots) in MCMV-infected control mice (+/+). B. Scatterplot analysis comparing MCMV-infected controls (+/+) and *Dicer^d/d^* mice. C–E. qRT-PCR data on selected MCMV-inducible genes (*Irf7, Ifit1* and *Oas1a*). The graphs show relative gene expression in controls (black spots; +/+) and *Dicer^d/d^* (white spots) spleen extracts. F. CXCL10 was measured in the serum of controls (white dots, +/+) and *Dicer^d/d^* mice (black dots) 36 hours p.i. G. IRF7 expression was analyzed by Western blot performed on protein extracts from MCMV-infected wild-type (+/+) and mutant mice. GAPDH expression serves a loading control.

Next, we searched for molecular defects that might account for the increased MCMV susceptibility of Dicer deficient animals. To this end, we performed a PCR macroarray analysis to monitor the effect of the *Dicer^d/d^* mutation on the expression of IFN-regulated genes following MCMV infection. We first analyzed the expression of genes in the IFN pathway in the spleen of animals at 3 d.p.i. As expected, we observed that in wild-type animals many (40/84) genes were induced upon MCMV infection (Fig. 6A), whereas in *Dicer^d/d^* mice the MCMV response appears to be globally deregulated (Fig. 6B). We validated these observations using qRT-PCR and confirmed that MCMV-induced genes such as *Irf7*, *Ifit1* and *Oas1a* are significantly reduced in Dicer deficient conditions (Fig. 6C-E). These results were confirmed with peritoneal macrophages isolated from control and mutant mice (Fig. S2). At the protein level, the expression of MCMV-induced CXCL10 in the serum of infected *Dicer^d/d^* mice was significantly reduced compared to wild-type animals (Fig. 6F). Similarly, the expression of MCMV-dependent IRF7 is diminished in protein extracts from Dicer mutant spleens compared to wt (Fig. 6G).

Globally, the picture emerging from our data (Fig. S3) indicates that in spleen and primary macrophages isolated from *Dicer^d/d^* mice, IFN-stimulated gene expression induced by MCMV infection is reduced. This indicates that, in the tight balance between repressors and activators that govern the efficient control of gene expression, a miRNA-dependent control of repressors could be an important facet of the transcriptional control of IFN-stimulated gene expression.

Because gene responses following acute viral (including MCMV) infection strongly depend on the activation of Toll-like Receptors (TLRs) [11], [12], a miRNA-dependent modulation of these factors might explain the above-described phenotype. However, we were unable to detect any significant decrease in type I IFN response after stimulation of wt and Dicer^d/d^ macrophages with several TLR ligands (data not shown).

Going more into the details of each gene present on the array, we observed that the transcriptional response to MCMV infection in primary macrophages is highly homogenous with one group of genes going down in mutant cells and the other remaining constant. In fact, we only observed two exceptions to this classification at 24 h post-infection: Psme2 (more induced in mutants than in controls), and Samsn1 (repressed in controls, and unaffected in mutant cells). Despite these two marginal cases, these experiments clearly indicate that Dicer reduction provokes a highly significant (p<0.0001, calculated at 24 h and 72 h post-infection, see Fig. S3) global 2-fold decrease in the expression of IFN-stimulated genes. Similar conclusions can be drawn from the analysis of whole spleens.

### 8. Profiling of cellular and viral miRNAs upon MCMV infection

To complete our analysis, we measured by PCR array the expression of miRNAs associated with inflammation/immunity upon MCMV infection of mice or peritoneal macrophages. In these two experiments, the results of the macroarray enable important observations. We found that several miRNAs were induced by MCMV in splenocytes of control mice (not shown). Among these upregulated miRNAs are miR-155 (4.3-fold), a well described actor in inflammatory reactions [25], and the TLR-dependent miR-147 (8-fold) [26], which has also been recently shown to be involved in the response to dengue virus [27]. In accordance with our repressor hypothesis described above, it is tempting to speculate that these MCMV-induced miRNAs could target repressor genes, thus permitting a rapid increase in IFN-stimulated gene expression. In Dicer mutant splenocytes, the induction of these miRNAs is impaired, which might lead to augmented repressor activity, and thus a diminished IFN response. We also note with interest that MCMV infection leads to the downregulation of certain miRNAs (miR-135b and miR-145 exhibit respectively 3.5-fold and 4.9-fold reduction), suggesting viral control of cellular miRNA production.

In MCMV-infected *Dicer^d/d^* peritoneal macrophages, we observed that although some miRNAs are induced, there is a highly significant decrease in the expression of certain miRNAs (Fig. S4). A quantification of this decrease (Fig. S4B) shows that the overall fold change is 0.26 in controls *vs*. −1.3 in *Dicer^d/d^* macrophages (p<0.0001).

**Figure 7:**
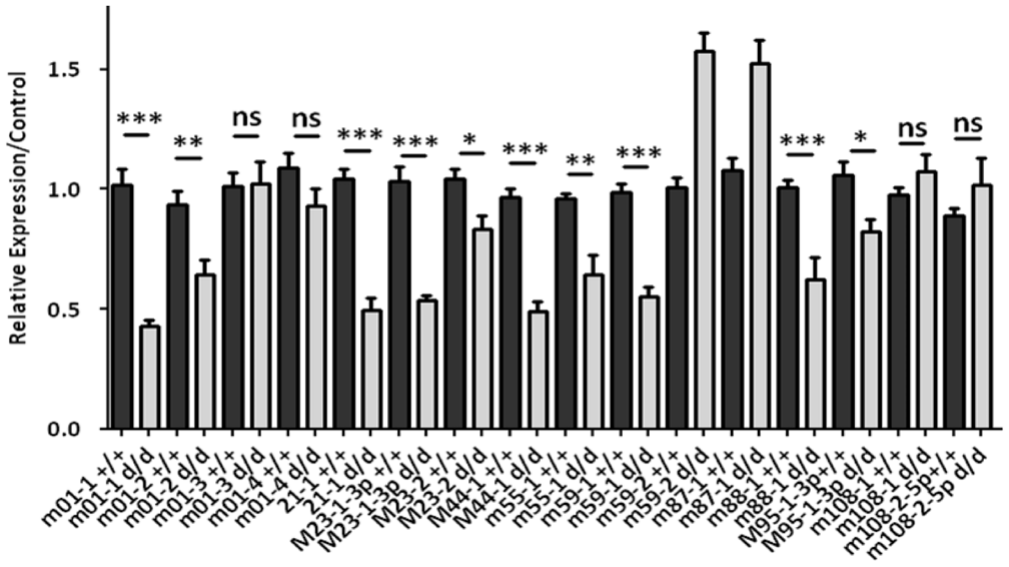
*Dicer^d/d^* mutation affects the expression of a subset of MCMV-encoded miRNAs in macrophages. Virally-derived miRNAs were quantified by qRT-PCR in MCMV-infected macrophages harvested from control (dark bars, +/+) or *Dicer^d/d^* mutant mice (light grey bars).

Finally, we also quantified by qRT-PCR 16 out of 18 MCMV-encoded miRNAs in spleens of MCMV-infected controls and Dicer-deficient mice. Ten viral miRNAs (62.5%) exhibited significantly reduced expression in mutant splenocytes (Fig. 7). Surprisingly, the expression of mcmv-miR-m59-2 and miR-m87-1 appeared augmented in splenocytes isolated from *Dicer^d/d^* mice. However, this seems to be due to a specificity issue since we also detected expression of miR-m59-2 in uninfected spleen cells (data not shown). Similar to our previous observations for cellular miRNAs, certain viral miRNAs (e.g. miR-m01-3 and m01-4) are expressed at the same level in wild-type and mutants. This asks the question as to the constraints that are required at the pre-miRNA level for Dicer maturation.

## Discussion

### 1. *In vivo* identification of functional miRNA/mRNA-targeted genes

As opposed to classical approaches where miRNA target predictions lead the way to identify relevant miRNA-target pairs, we chose here to analyze the phenotype of Dicer mutants to identify potential miRNA targets, and then, to use *in silico* analyses and reporter-based assays for confirmation. As an illustration of this procedure, Cxcl10 was selected because altered expression of this chemokine is associated with inflammatory defects and viral susceptibility [28]. By inhibiting the endogenous human miR-21 (which is 100% identical to the *Mus musculus* ortholog) in Hela cells, we found that the expression of CXCL10 was regulated by this miRNA (Fig. 3). Interestingly, we noticed the occurrence of 2 SNPs (rs8878 and rs3921) that may affect miRNA binding sequences in the human Cxcl10 3’UTR, as predicted by a new web-based tool, MicroSNiPER [29]. Furthermore, we observed miR-21 induction in controls compared to MCMV-infected splenocytes (2.3 fold) and macrophages (1.8 fold). However, similar induction rates were obtained in tissues harvested from Dicer mutant animals upon viral infection.

Many reports have described the influence of viral infection on individual cellular miRNA expression (reviewed in [4]). Our effort to concur in a wider description of miRNA involvement in host-pathogen interactions is in agreement with previous data showing up-regulation of cellular miRNAs such as miR-155 and miR-147, which are involved in the control of inflammatory responses [25], [26]. But our microarray analysis unveils additional miRNAs that may be essential modulators of this process in acute MCMV infection. Indeed, miR-182, miR-183 and miR-31 are strongly induced upon MCMV *in vivo* infection, and interestingly two of these, miR-182 and 183 are also induced upon human CMV infection of fibroblasts [30]. In addition, the same set of miRNAs was shown to be overexpressed in splenocytes of Lupus-prone mice [31], which is in agreement with our model predicting that these miRNAs might target transcriptional repressors of IFN-stimulated genes.

It would be of interest to perform a thorough analysis of the promoters of MCMV-inducible miRNAs to identify potential IFN-responsive elements. This would then allow the reconstitution of a complex network of interactions between miRNAs and viral infection.

### 2. Induction of IFN-dependent genes upon viral infection requires repressor release

**Figure 8:**
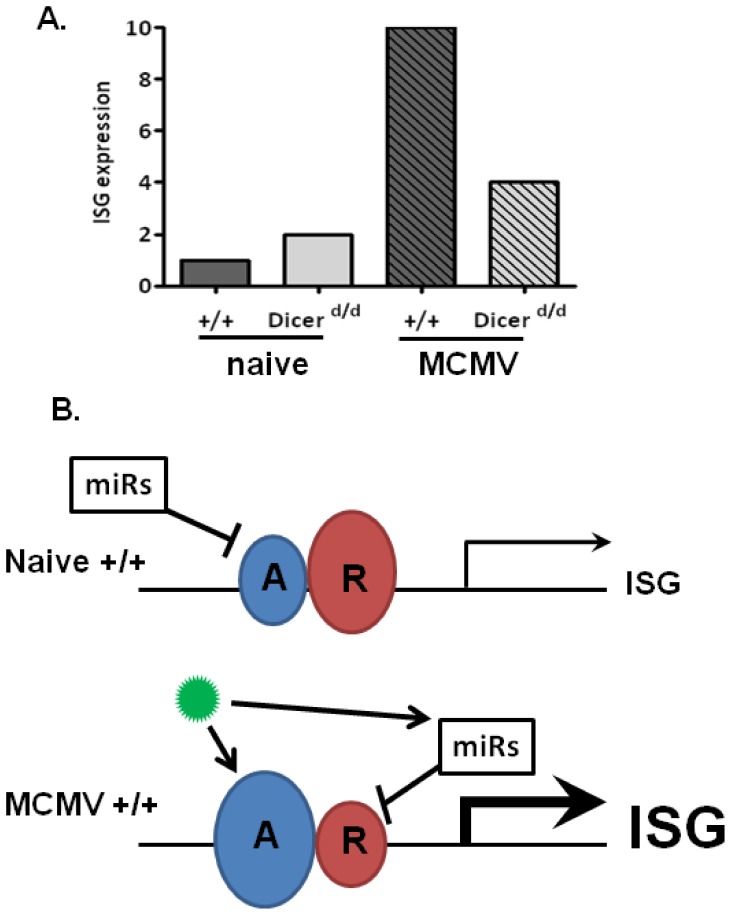
Model for miR-dependent control of IFN-regulated genes. A. Schematic representation of our macroarray data. Interferon-stimulated genes (ISG) expression is indicated for controls (+/+, dark grey) and *Dicer^d/d^* (light grey) cells in naïve or MCMV-stimulated conditions. B. In naïve control (+/+) cells, repression of ISGs is secured by miRNA-mediated repression exerted on transcriptional activators (in blue); on the contrary, rapid induction of ISGs upon virus (depicted in green) infection is permitted by MCMV-induced miRNA-mediated release of repressors (in red) along with virally-induced transcriptional induction of activators.

Although the protective role of type I IFN against viral infections has been intensively studied for decades [32], [33], a thorough understanding of the transcriptional program engaged upon viral infection is far from complete. It is now well established that the panel of IFN-stimulated genes (ISGs) is both cell and virus dependent, which illustrates the complexity of the so-called ‘IFN response’. Here, we addressed the potential role of miRNAs in the control of IFN-dependent, MCMV-regulated genes. As schematized in Fig. 8A, we observe a Dicer-dependent increase in IFN-response gene expression under naive conditions, whereas the opposite was observed in MCMV infected samples. Given the inhibitory effect of miRNAs, we suggest that miRNAs limit the level of transcriptional activators under steady-state conditions to ensure repressor dominance, thus preventing leakage of ISGs that could trigger adverse reactions (Fig. 8B). As inferred from our data, STAT1 is a likely miRNA-dependent activator (Fig. S1) and we are currently testing this hypothesis. We also keep in mind that miRNAs could indirectly impact upon the expression of Interferon-responsive genes. For instance, transcription elongation PAF1, which selectively mediates antiviral genes response [34] could also be under miRNA-dependent regulation.

Next, our model also predicts that following viral infection miRNA-dependent repressor release from target genes enables rapid gene activation, but upon low miRNAs conditions in *Dicer^d/d^* mice, such repressors (e.g TRIM28 [35]) are overexpressed, hence the lower IFN response. An efficient and harmless for the host antiviral program requires tight control of the inflammatory response. This has been demonstrated in the case of TLR-dependent TNF-α synthesis in which repressors such as IRAK-M or SOCS1 [36] appear essential in the post-induction turn-off. Similarly, Type I IFN-dependent expression must also be under strict fine-tuning, but the underlying mechanisms are still largely unknown [37].

In addition, our macroarray analysis provides a unique and comprehensive picture of IFN-dependent gene induction upon MCMV infections. These data confirmed the importance of master factors such as IRF7, which is strongly induced by MCMV both *in vivo* and *ex vivo*. Interestingly, we also noted MCMV-dependent down modulation of several genes (i.e. *Irf2*, *Irf3*, *Irf5*), which reveals potential virus hijacking. Recent demonstration that Kaposi's sarcoma herpesvirus encodes a viral IRF3 that inhibits cellular IRF5 strengthens this suggestion [38].

### 3. miRNA: antiviral players in vertebrates? What our model teaches us

An important objective of our work was to measure the importance of miRNAs as defensive tools against MCMV infection. A large amount of data has demonstrated that *Dicer* targeted inactivation impairs immune functions, but a global *in vivo* evaluation of the roles of miRNAs in antiviral defense in a relevant and authentic animal model had so far not been reported. A previous report had described increased susceptibility to VSV infection in Dicer mutant mice [6]. However, a major weakness of this model was its lack of physiological relevance, since VSV is not a natural pathogen of mice. To circumvent these restrictions, we analyzed IFN-stimulated genes and inflammation/immunity-related miRNA expression in Dicer-deficient mice, both in naive conditions and upon acute MCMV infection. We also focused on herpesviruses because these pathogens express their own set of miRNAs. The existence of these molecules, likely hijacked by viruses for their own benefit, is suggestive that the miRNA machinery actively participates in innate antiviral responses. Indeed, MIC-B is targeted by HCMV-derived miRNAs, which allow the pathogen to escape NK-cell-mediated killing [39]. Our own data somehow contradict this model in which viral miRNAs would be considered as pathogenicity factors. Indeed, we observe lower survival in Dicer-deficient animals (Fig. 4), whereas diminishing viral miRNA maturation would expect to decrease MCMV pathogenicity. On the other hand, *Dicer ^d/d^* mutant mice are also characterized by diminished cellular miRNAs synthesis, which lowers inflammatory response upon MCMV infection, hence increasing host susceptibility. It is therefore difficult to weigh the exact contribution of pathogenic viral miRNAs *vs*. protective cellular miRNAs in our complex *in vivo* model, but our survival data nevertheless suggest that cellular miRNAs prevail over viral molecules. This observation may have important repercussions in human health, where null mutants are unlikely, but variable DICER levels in the population could account for differences in susceptibility to pathogens among individuals.

Finally, our *in vivo* approach in mice indicates that Dicer reduction induces only limited viral susceptibility compared to mutants affecting IFN signaling, as noted previously [40]. This contrasts with the situation in plants [41] or Drosophila [42], whose defense mechanisms rely solely on RNA interference, and in which severe phenotypes appear under viral infection of Dicer mutants. Therefore, our *in vivo* data suggest that a fine-tuned IFN response to a viral pathogen in vertebrates requires a carefully balanced arsenal of cellular miRNAs.

## Supporting Information

Figure S1 **STAT1-dependent genes are induced in** ***Dicer^d/d^*** **mice.** The difference in gene expression (fold regulation) between control and *Dicer^d/d^* splenocytes was measured; genes were separated according to the presence of STAT1-binding motifs in their promoters. ** p = 0.0016.
(TIF)

Figure S2 **Dicer^d/d^ mutation impairs IFN-stimulated genes induction upon MCMV infection of isolated macrophages.** A. Scatterplot showing relative gene expression between non-infected (NI) and MCMV-stimulated macrophages from wild-type (+/+) mice. B. Scatterplot showing relative gene expression between MCMV-infected macrophages from control (+/+) mice compared to cells harvested from *Dicer^d/d^* animals. C-E. RT-qPCR data showing lower expression of selected genes (irf7, Oas1a and Isg20) in cells isolated from *Dicer^d/d^* mice compared to macrophages from controls (+/+).
(TIF)

Figure S3 ***Dicer^d/d^*** **mutation impairs IFN-stimulated genes induction upon MCMV infection; statistical analysis.** A. Histogram showing relative expression of IFN-dependent genes (fold change between non-infected *vs*. MCMV-stimulated) in macrophages from controls (black bars) and *Dicer^d/d^* (white bars) mice 24 hours p.i. B. Similar analysis performed 72 hours p.i. C. Similar analysis performed in splenocytes 3 days upon *in vivo* MCMV infection of mice.
(TIF)

Figure S4 **miRNAs expression is strongly reduced in MCMV-infected macrophages harvested from** ***Dicer^d/d^*** **mice.** A. Pie chart representing the proportion of overexpressed (green), repressed (blue) and unaffected miRNAs involved in immunity/inflammation in MCMV-stimulated control (+/+) macrophages and *Dicer^d/d^* mutants. B. Histogram showing relative expression (Fold change between naïve and MCMV-infected) of miRNAs in control (+/+, dark bar) and *Dicer^d/d^* (light grey bar) macrophages.
(TIF)
