# Supplementary figures and images for: Deregulation of Type I IFN-Dependent Genes Correlates with Increased Susceptibility to Cytomegalovirus Acute Infection of Dicer Mutant Mice
Source: PLoS One. 2012 Aug 20;7(8):e43744. doi: 10.1371/journal.pone.0043744 (PMC3423365; doi:10.1371/journal.pone.0043744)

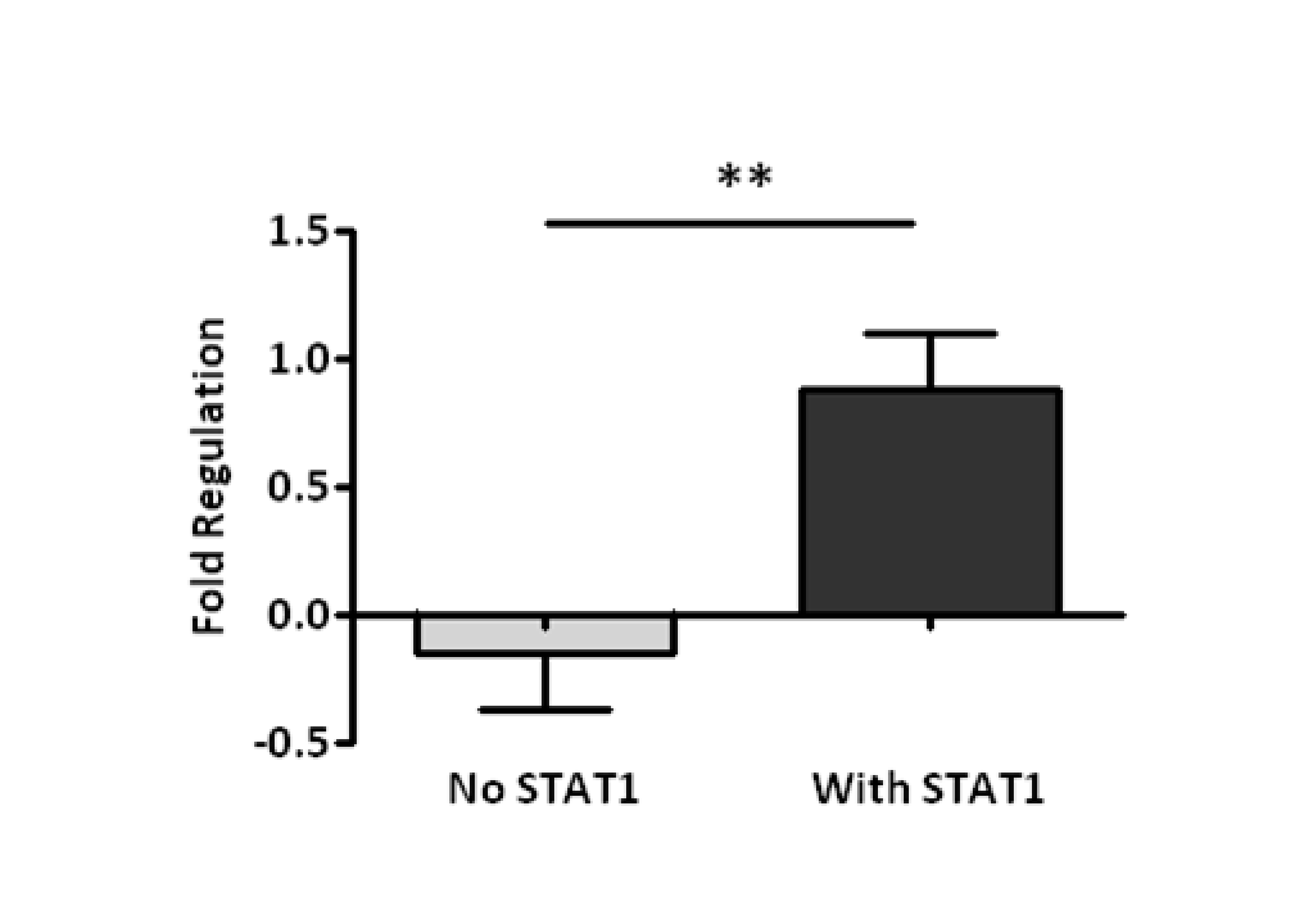

Supplement: Figure S1 — STAT1-dependent genes are induced in Dicerd/d mice. The difference in gene expression (fold regulation) between control and Dicerd/d splenocytes was measured; genes were separated according to the presence of STAT1-binding motifs in their promoters. ** p = 0.0016. (TIF) [file pone.0043744.s001.tif]

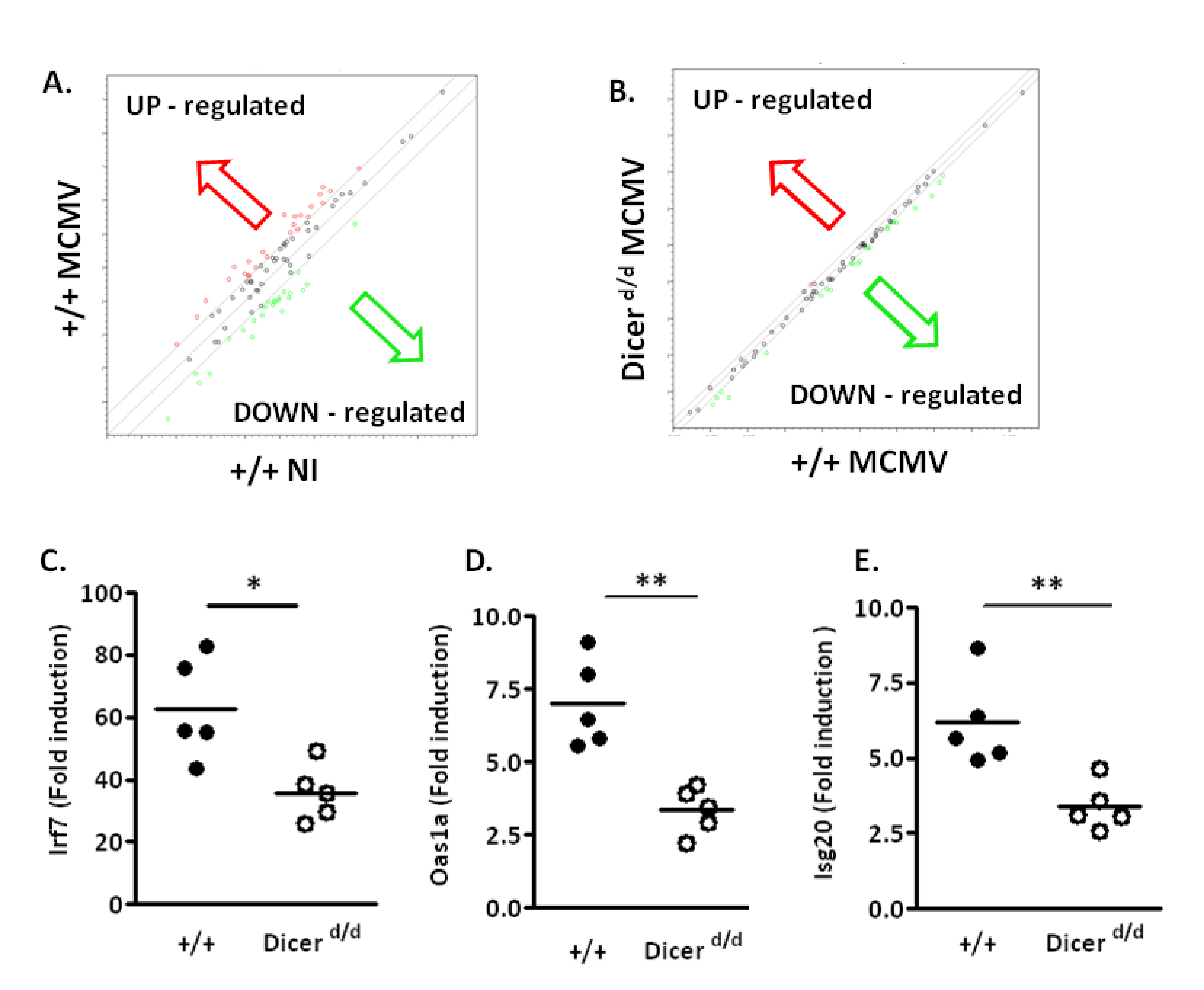

Supplement: Figure S2 — Dicerd/d mutation impairs IFN-stimulated genes induction upon MCMV infection of isolated macrophages. A. Scatterplot showing relative gene expression between non-infected (NI) and MCMV-stimulated macrophages from wild-type (+/+) mice. B. Scatterplot showing relative gene expression between MCMV-infected macrophages from control (+/+) mice compared to cells harvested from Dicerd/d animals. C-E. RT-qPCR data showing lower expression of selected genes (irf7, Oas1a and Isg20) in cells isolated from Dicerd/d mice compared to macrophages from controls (+/+). (TIF) [file pone.0043744.s002.tif]

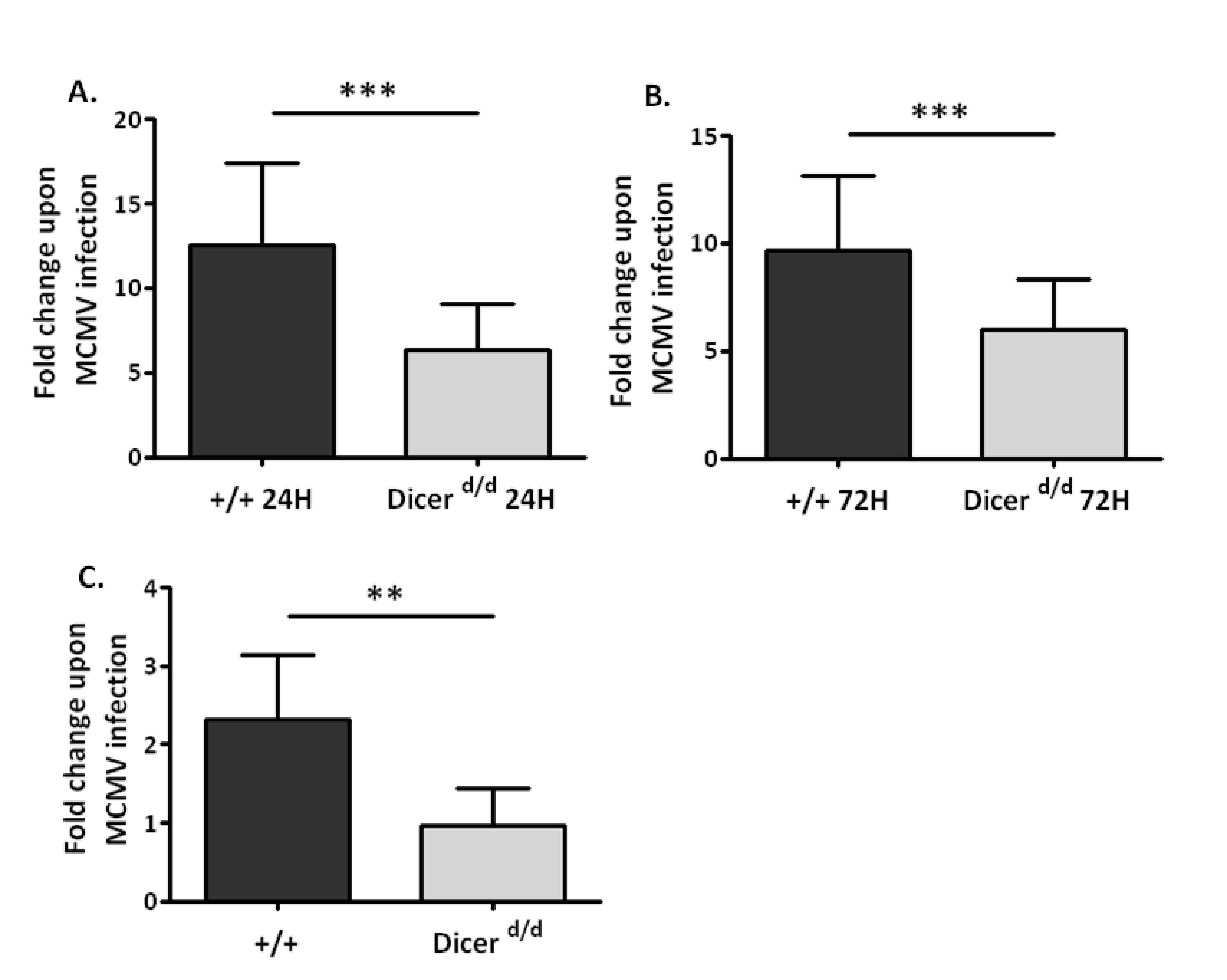

Supplement: Figure S3 — Dicerd/d mutation impairs IFN-stimulated genes induction upon MCMV infection; statistical analysis. A. Histogram showing relative expression of IFN-dependent genes (fold change between non-infected vs. MCMV-stimulated) in macrophages from controls (black bars) and Dicerd/d (white bars) mice 24 hours p.i. B. Similar analysis performed 72 hours p.i. C. Similar analysis performed in splenocytes 3 days upon in vivo MCMV infection of mice. (TIF) [file pone.0043744.s003.tif]

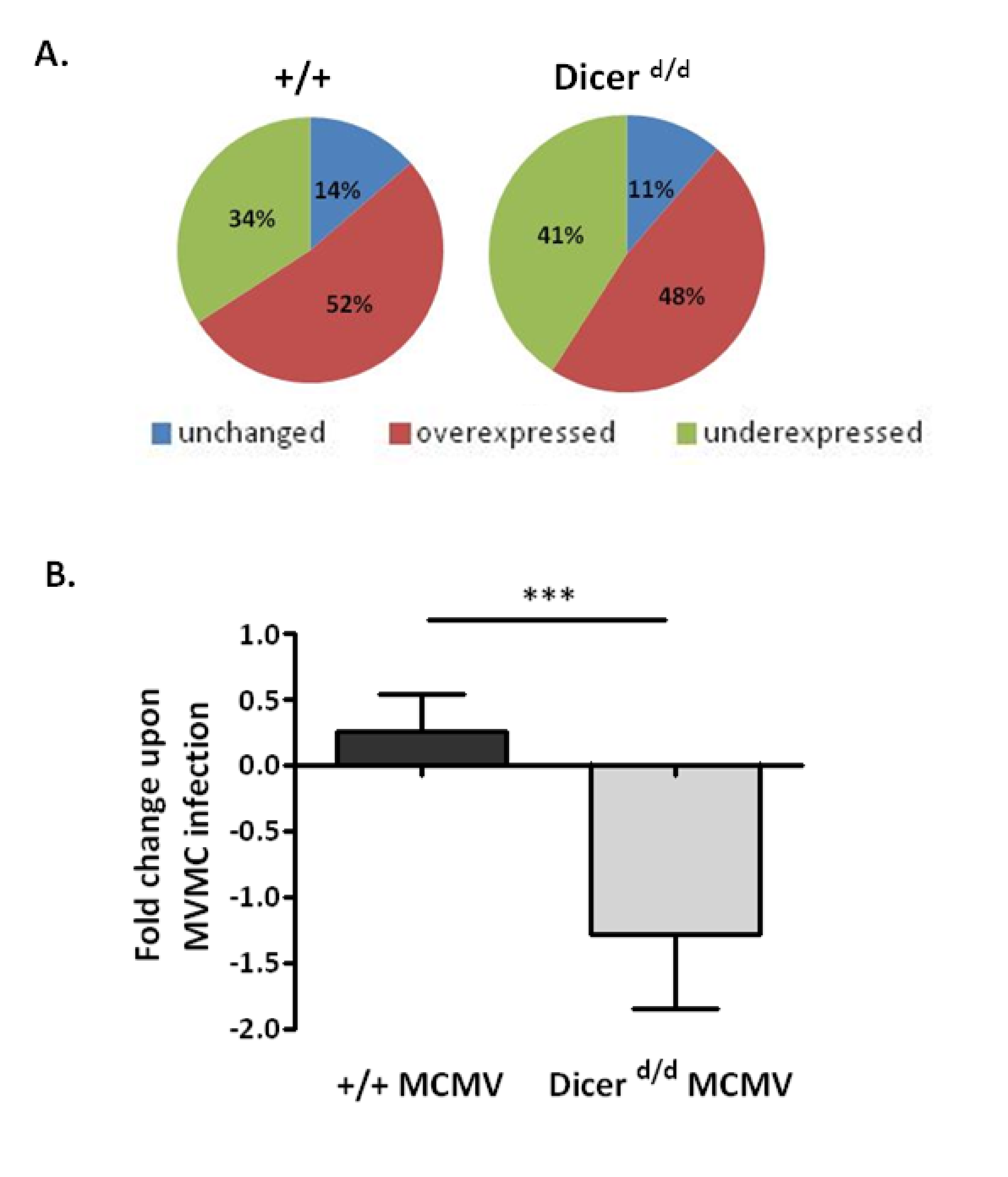

Supplement: Figure S4 — miRNAs expression is strongly reduced in MCMV-infected macrophages harvested from Dicerd/d mice. A. Pie chart representing the proportion of overexpressed (green), repressed (blue) and unaffected miRNAs involved in immunity/inflammation in MCMV-stimulated control (+/+) macrophages and Dicerd/d mutants. B. Histogram showing relative expression (Fold change between naïve and MCMV-infected) of miRNAs in control (+/+, dark bar) and Dicerd/d (light grey bar) macrophages. (TIF) [file pone.0043744.s004.tif]
